# 3D mapping reveals network-specific amyloid progression and subcortical susceptibility in mice

**DOI:** 10.1038/s42003-019-0599-8

**Published:** 2019-10-04

**Authors:** Rebecca Gail Canter, Wen-Chin Huang, Heejin Choi, Jun Wang, Lauren Ashley Watson, Christine G. Yao, Fatema Abdurrob, Stephanie M. Bousleiman, Jennie Z. Young, David A. Bennett, Ivana Delalle, Kwanghun Chung, Li-Huei Tsai

**Affiliations:** 1 The Picower Institute for Learning and Memory, Department of Brain and Cognitive Sciences, Massachusetts Institute of Technology, Cambridge, MA USA; 2 Institute for Medial Engineering and Science (IMES), MIT, Cambridge, MA USA; 3 Rush Alzheimer’s Disease Center, Rush University Medical Center, Chicago, IL USA; 4 Department of Pathology and Laboratory Medicine, Boston University School of Medicine, Boston, MA USA; 5 Department of Chemical Engineering, MIT, Cambridge, MA USA

**Keywords:** Alzheimer's disease, Neural circuits

## Abstract

Alzheimer’s disease (AD) is a progressive, neurodegenerative dementia with no cure. Prominent hypotheses suggest accumulation of beta-amyloid (Aβ) contributes to neurodegeneration and memory loss, however identifying brain regions with early susceptibility to Aβ remains elusive. Using SWITCH to immunolabel intact brain, we created a spatiotemporal map of Aβ deposition in the 5XFAD mouse. We report that subcortical memory structures show primary susceptibility to Aβ and that aggregates develop in increasingly complex networks with age. The densest early Aβ occurs in the mammillary body, septum, and subiculum- core regions of the Papez memory circuit. Previously, early mammillary body dysfunction in AD had not been established. We also show that Aβ in the mammillary body correlates with neuronal hyper-excitability and that modulation using a pharmacogenetic approach reduces Aβ deposition. Our data demonstrate large-tissue volume processing techniques can enhance biological discovery and suggest that subcortical susceptibility may underlie early brain alterations in AD.

## Abstract (web summary)

Rebecca Canter et al. use the SWITCH technique for immunolabeling whole brain to show that core regions of the Papez memory circuit are susceptible to Aβ accumulation in mice. They find that Aβ accumulation in the mammillary body is linked to neuronal hyper-excitability and amyloid deposition can be reduced using a pharmacogenetics approach.

## Introduction

Cognitive impairments attributable to Alzheimer’s disease (AD) will affect millions of individuals in the next decade, however, the etiology of the disease remains largely unknown^1^. The results from decades of research support the hypothesis that the accumulation of toxic amyloid-beta peptides (Aβ) in the brain contributes to the onset and progression of Alzheimer’s dementia^2–4^. The amyloid hypothesis was initially based on the discovery that mutations in the Aβ precursor protein (APP) and its processing enzymes cause autosomal dominant, inherited, familial Alzheimer’s dementia (FAD)^5^. Subsequent preclinical studies demonstrating that Aβ peptides induce synaptic loss and neuronal death in vitro and in vivo have further established that Aβ is acutely toxic to the neural substrate. In addition to cellular harm, amyloidosis in murine models of AD contributes to AD-like memory impairments, hippocampal synaptic loss, and electrophysiological alterations—changes that are also observed in human patients with amyloid^6–8^. Together, the preclinical and genetic data suggest that Aβ directly impacts the neurodegeneration that is observed FAD^9–11^ and also provide a basis for understanding the etiology of the sporadic form of the disease. Despite the link between Aβ toxicity and neurodegeneration in multiple forms of AD, the precipitating events that trigger Aβ accumulation, deposition, and progression remain unclear.

One impediment to understanding the relationship between Aβ deposition and the onset of AD has been identifying the brain regions that are most vulnerable to Aβ plaques. The first studies using postmortem brain sections from AD patients’ brains suggested that initial accumulation of Aβ occurs in the neocortex with subsequent spread of aggregates to deeper structures implicated in learning and memory^12^. However, due to the use of postmortem specimens, these reports could not document Aβ deposition over time prior to the development of AD dementia. Recent advances in positron emission tomography (PET) imaging have enabled longitudinal human Aβ-imaging studies that confirm the importance of cortical Aβ in the diagnosis and prediction of progression of cognitive impairment^13,14^, although they have largely not examined deeper structures for Aβ load and spread^14,15^. Intriguingly, while cortical Aβ correlates with dementia status in patients^16,17^, many cognitively healthy individuals also have high levels of cortical Aβ^18^. A recent paper using PET staging showed a pattern of Aβ progression that was constant across individuals, regardless of clinical diagnosis, consistent with previous postmortem studies^19^. In addition, patients diagnosed with non-Alzheimer’s dementia-related neurodegenerative diseases, like Parkinson’s, showed regional Aβ vulnerability that correlates with cognitive and motor impairments^20^. This discrepancy between cortical accumulation and cognitive impairment suggests that a systematic and comprehensive approach to studying Aβ deposition may reveal unexplored Aβ-related changes in the brain that contribute proximally to memory-related alterations in Alzheimer’s dementia^21^.

Increasing evidence suggests that Aβ affects distributed memory networks in AD and that dysfunction in one or more of these networks may underlie cognitive decline. However, there are inconsistencies in these observations. Neuroimaging studies have revealed structural and functional alterations in high-level cortical networks like the default-mode network (DMN)^15,22^, while other recent findings show core alterations in fundamental deep memory structures that are part of the limbic system^8,23–25^. Although the networks underlying memory and those implicated by neuroimaging studies somewhat overlap, there are distinct brain areas and functions that the networks do not share. Because of this discrepancy, it remains uncertain where the most critical early network alterations occur in AD. Furthermore, the question of whether subcortical areas show differential susceptibility to cortical regions remains unexplored in most human neuroimaging studies.

One aspect that likely contributes to the difficulty discerning network alterations in AD is the challenge of staging patient disease progression. The complexity arises from the number of disease components that affect cognitive status, including variable pathological loads across patients^21^, as well as the socioeconomic, lifestyle, and genetic factors that influence AD^26^. To overcome the inherent variability in and technical limitations of studying human disease, in this work we started by capitalizing on the temporal precision of murine models to map Aβ progression at high-resolution throughout the brain of transgenic 5XFAD mice, which harbor five familial AD mutations—three in human *APP* and two in human *PSEN1*^27,28^. We use optimized techniques for whole-brain System-Wide Control of Interaction Time and kinetics of Chemicals (SWITCH) immunolabeling^29^ to create the unbiased and comprehensive map of Aβ deposition at high spatial and temporal resolution. Our observations reveal subcortical susceptibility to Aβ deposition in several regions of the Papez circuit, such as the dorsal subiculum, septum, and mammillary body. While other studies have implicated the subiculum in early AD, we were surprised to learn that the mammillary body, an area important in anterograde memory and spatial navigation, also may be vulnerable. We show that the MB neurons undergo functional alterations concurrent with early aggregate development and that modulating neural activity to counteract the functional changes, we observed lessens the Aβ burden in the mouse model. Together, our data suggest that the MB, as part of the Papez circuit, is a relevant and susceptible subcortical hub in the development of AD.

## Results

### Optimized SWITCH enables whole-brain immunolabeling

**Fig. 1:**
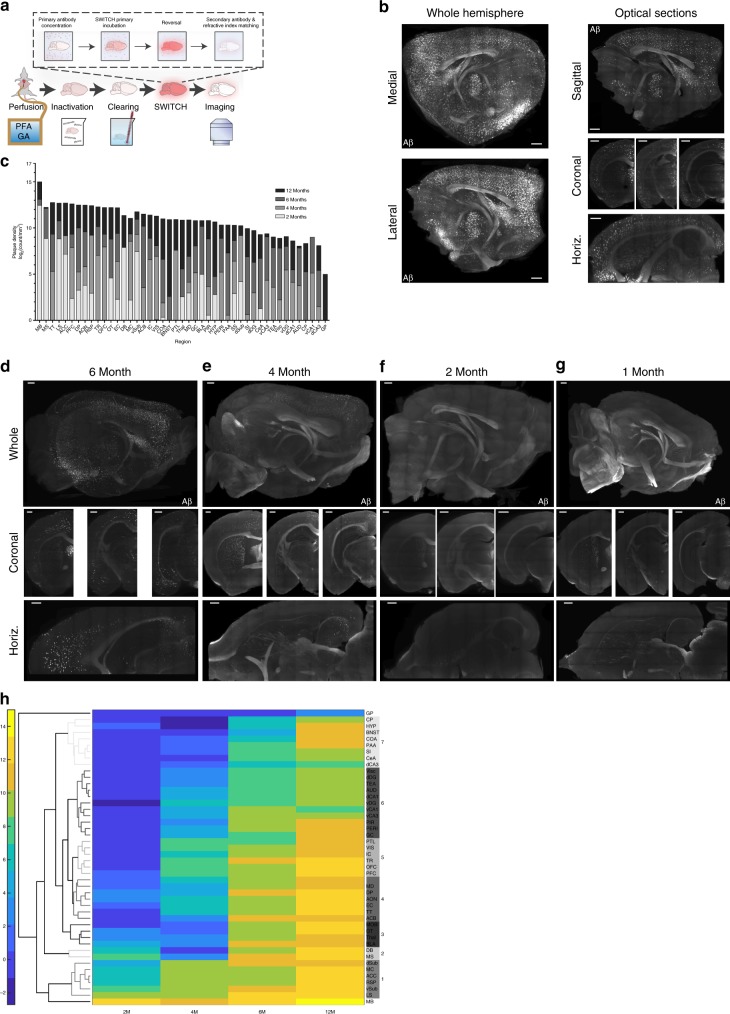
SWITCH labeling reveals Papez circuit vulnerability and network propagation of amyloid pathology. **a** Schematic of the System-Wide Control of Interaction Time and kinetics of Chemicals (SWITCH) protocol. **b** Representative images of amyloid labeling in a brain from a 12-month-old mouse. Whole hemisphere 2D images of the 3D-rendered brain from the medial and lateral view. Additional representative optical sections showing the sagittal distribution, horizontal distribution, and three coronal sections. Scale bars 1000 μm. **c** Amyloid density plot of average amyloid density for each age time point. *N* = 2 or 3/group. Log2-transformed deposit count/mm3of tissue. The data plotted in rank-order of 12-month animal density. Six-, 4-, and 2-month data overlaid on top of 12 M data for comparison. *N* = 1 (4 M); *N* = 2 (2 M, 6 M), *N* = 3 (12 M) independent biological samples. **d**–**g** Representative images of amyloid labeling in brains from (**d**) 6-, (**e**) 4-, (**f**) 2-, (**g**) 1-month-old mice. Scale bars 500 μm. **d** There is significant amyloid deposition in cortex, as well as aggregates in the hippocampus, amygdala, and other limbic structures in 6-month-old brains. **e** Representative images from 4-month-old brains show significant accumulation in the default-mode-related frontal cortex, and regions that are part of the Papez circuit (e.g., septum, subiculum, mammillary body). **f** Optical sections from 2-month-old brains showing sparse amyloid labeling, except for accumulation in the mammillary body, septum, and subiculum. **g** In 1-month-old brains, only a few small deposits can be seen in the subiculum and mammillary body. **h** Hierarchical clustering of log2-transformed average density data by region. Euclidean distance, average linkage. Scale bar is not symmetric around 0. Optimal leaf order enabled, which plots most similar groups nearest each other in the graph. Colored branches represent groups labeled on right *y-*axis

For a comprehensive and spatially unbiased, temporally precise map of amyloidosis in a murine model of AD, we needed to label intact tissue specimens that we could image at a cellular scale. This would enable us to create a specific pathological map like with traditional histology, but without the loss of critical information to the size limitations and directional segmentations that are inherent in tissue-sectioning studies. We chose to use SWITCH techniques because they enable homogenous, whole-brain immunolabeling using readily accessible laboratory reagents^29^. Although the SWITCH techniques are widely applicable to many antibodies and target proteins^29^, we discovered that we needed to further refine the buffer systems to achieve homogenous whole-brain labeling with our chosen antibodies. In our optimized SWITCH system (Fig. 1a), we modulate the pH and ionic strength of the buffers to control antibody–antigen binding kinetics^30^. In the first step of our new protocol, we use a high-pH and high ionic- strength buffer to slow the binding reaction. This modulation allows antibodies to penetrate deep into the sample passively, regardless of the antigenic content in the tissue. In the second step, we titrate the buffer back to a physiological pH and ionic strength to re-enable antibody binding. By employing this system, we achieved homogenous labeling throughout thick tissue sections using an antibody to myelin basic protein (MBP), an extremely abundant antigen (Supplementary Fig. 1A). In addition, we reduced signal intensity changes through the thickness of the tissue (Supplementary Fig. 1B) and created a signal attenuation profile consistent with light loss through the sample, rather than due to non-homogenous labeling within the specimen (Supplementary Fig. 1C).

Despite showing the protocol worked with MBP antibodies in thick tissue sections, we needed to ensure that our optimized SWITCH was compatible with our chosen Aβ antibody. To test this, we used 12–18-month-old 5XFAD mouse brains. We chose this age range based on previous reports that demonstrated wide-spread aggregate accumulation and substantial local Aβ burden developed by that time^28,31^. Applying the optimized SWITCH protocols to the 5XFAD brains, we observed homogenous immunolabeling of Aβ aggregates throughout the samples from aged mice, including identifiable labeling in the centermost areas (Supplementary Movie 1). Qualitative analyses revealed accumulation of Aβ was evident in some unexpected subcortical regions in addition to the previously reported cortical and hippocampal regions (Supplementary Fig. 1D–F). These proof-of-concept samples confirmed that our SWITCH-based large tissue volume technique was a reliable tool to analyze intact specimens and suggested that we might be able to uncover interesting biology by using the new technique to investigate regional amyloid burden^32^.

### Spatiotemporal analyses reveal papez circuit vulnerability

To quantify regional burden more comprehensively and at a higher spatial resolution, especially in the interesting subcortical areas, we carried out the SWITCH procedure on brains from a cohort of 12-month (12 M)-old 5XFAD mice (Fig. 1b; Supplementary Movie 2D). Region-specific quantification from white matter-based hand annotation of three-dimensional (3D) images showed substantial Aβ deposition throughout the brains and confirmed accumulation in AD-associated areas (Fig. 1c). The regions harboring Aβ included the retrosplenial cortex (RSP), hippocampus (HPC), subiculum (SUB), and anterior cingulate cortex (ACC), and prefrontal cortex (PFC). Importantly, deposits were largely absent from areas that appear less vulnerable to Aβ aggregation in human AD brains, such as the caudoputamen (CP) and globus pallidus (GP). Differences in Aβ levels across regions were significant (D’Agostino and Pearson normality test; K2 = 94.61, *p* < 0.0001, passed normality = no; Friedman test; *Q* = 114.2, *p* < 0.0001), which suggests that the peptide accumulates within particular brain areas and does not deposit unvaryingly across the brain. Unexpectedly, the highest aggregate density by rank in the 12 M animals appeared within specific subcortical regions, namely the medial nucleus of the mammillary body (MB) and septum (SEPT).

The regional differences in Aβ and unexpected high density in small subcortical nuclei might develop because aggregates in the 5XFAD animals arise uniformly throughout the brain over time, thus making these smaller structures appear denser. Alternatively, the high-density regions could show unique vulnerability to form aggregates in conditions of elevated Aβ, leading to earlier or more substantial deposition. To observe which model better explains the amyloid distribution, we applied our optimized SWITCH protocol to map the sequence of amyloid deposition back to the earliest time points by analyzing brains from 6, 4, 2, and 1-month (6 M, 4 M, 2 M, 1 M, respectively)-old 5XFAD mice (Supplementary Movie 2A–C). We quantified Aβ density in many regions across multiple animals at each time point in a similar manner as in the 12 M brains, and observed increasingly specific aggregation with decreasing age in a pattern that was consistent across individuals. At 6 M, 5XFAD brains displayed region-specific aggregation (Fig. 1d), with areas harboring significantly different densities of amyloid (Fig. 1c; D’Agostino and Pearson normality test; K2 = 70.12, *p* < 0.0001, passed normality = no; Friedman test; *Q* = 68.56, *p* = 0.0171). To better understand the brain areas that contribute to region-specific aggregation in our data, we performed hierarchical clustering analysis on the log2 transform of the regional data averages from each age cohort (Fig. 1h, Euclidean distance, average linkage). The clustering revealed a number of groups, each of which contained areas that function as nodes in specific cognitive networks (Table 1). At 6 M, the hippocampus, olfactory, and corticolimbic (e.g., basolateral amygdalar complex (BLA), piriform cortex (PIR), entorhinal cortex (EC)) systems first show appreciable levels of Aβ (Table 2). This result was particularly interesting because the clustering analysis is based solely on aggregate numbers and it found that the density of Aβ is relatively uniform within functional networks, and also unbiasedly identified circuits that underlie behaviors and cognitive functions known to show alterations in early Alzheimer’s dementia^33^.

**Table 1:** Regional group assignments from Fig. 1

Group	Regions	Primary network	Secondary networks
-	MB	Papez	
1	vSub, dSub, LS, RSP, ACC, MC	Papez	Default mode
2	MS, DB	Cholinergic	
3	BLA, Thal, OT, MOB	Limbic	Olfactory
4	EC, ACB, MD, SS,DP, TT, AON	Cortex	Olfactory, basal ganglia
5	PFC, OFC, PTL, IC, VIS, TR	Cortex	
6	dDG, dCA1, vDG, vCA1, vCA3, Peri, AUD, TEA, Pir, GC, Visc	Hippocampus	Cortex
7	dCA3, CeA, SI, PAA, COA, BNST, HYP, CP	Limbic	Straitum
-	GP	Striatum	

*ACC* anterior cingulate cortex, *RSP* retrosplenial cortex, *vSub* ventral subiculum, *LS* lateral septum, *dSub* dorsal subiculum, *MC* motor cortex, *DB* diagonal band, *MS* medial septum, *ACB* nucleus accumbens, *PIR* piriform cortex, *MD* midbrain, *Thal* thalamus, *BLA* basolateral amygdala, *OT* olfactory tubercle, *PERI* perirhinal and ectorhinal corticies, *GC* gustatory cortex, *IC* insular cortex, *VIS* visual cortex, *PTL* posterial parietal association areas, *SS* somatosensory cortex, *DP* dorsal peduncular area, *AON* anterior olfactory nucleus, *EC* entorhinal cortex, *PFC* prefrontal cortex, *OFC* orbitofrontal cortex, *TR* postpiriform transition area, *TT* tenia tecta, *dDG* dorsal dentate gyrus, *Visc* visceral cortex, *TEA* temporal association areas, *AUD* auditory cortex, *dCA1* dorsal CA1, *vDG* ventral dentate gyrus, *vCA1* ventral CA1, *vCA3* ventral CA3, *dCA3* dorsal CA3, *CP* caudoputamen, *BNST* bed nucleus of the stria terminalis, *HYP* hypothalamus, *CeA* centromedial amygdalar nuclei, *SI* substantia innominata, *PAA* piriform amygdalar area, *COA* cortical amygdalar area

Primary and secondary network assignment based on the regions in the group and major networks that appear in literature searches associated with each region

**Table 2:** Group-network data summarized from Fig. 1

Network	Groups	Age of Aβ appearance
Papez	MB, 1, 3	2 months
Cholinergic	1, 2	2 months
Default mode	1, 4	4 months
Sensorimotor cortex	1, 5, 6	4 months
Olfactory	3, 4, 6	6 months
Extended limbic	3, 4, 5, 7	6 months
Hippocampus	6, 7	6 months
Straitum	7, GP	12 months

*ACC* anterior cingulate cortex, *RSP* retrosplenial cortex, *vSub* ventral subiculum, *LS* lateral septum, *dSub* dorsal subiculum, *MC* motor cortex, *DB* diagonal band, *MS* medial septum, *ACB* nucleus accumbens, *PIR* piriform cortex, *MD* midbrain, *Thal* thalamus, *BLA* basolateral amygdala, *OT* olfactory tubercle, *PERI* perirhinal and ectorhinal corticies, *GC* gustatory cortex, *IC* insular cortex, *VIS* visual cortex, *PTL* posterial parietal association areas, *SS* somatosensory cortex, *DP* dorsal peduncular area, *AON* anterior olfactory nucleus, *EC* entorhinal cortex, *PFC* prefrontal cortex, *OFC* orbitofrontal cortex, *TR* postpiriform transition area, *TT* tenia tecta, *dDG* dorsal dentate gyrus, *Visc* visceral cortex, *TEA* temporal association areas, *AUD* auditory cortex, *dCA1* dorsal CA1, *vDG* ventral dentate gyrus, *vCA1* ventral CA1, *vCA3* ventral CA3, *dCA3* dorsal CA3, *CP* caudoputamen, *BNST* bed nucleus of the stria terminalis, *HYP* hypothalamus, *CeA* centromedial amygdalar nuclei, *SI* substantia innominata, *PAA* piriform amygdalar area, *COA* cortical amygdalar area

Ordered by group average (MB–0, GP–8). Age of appearance assigned based on log-scale values for majority of regions > 4

To further refine our understanding of the sequence of Aβ deposition, we next looked at 4 M-aged brains in the 5XFAD predictable, genetic murine model (Fig. 1e). Importantly, at 4 M, the data continue to demonstrate an increasingly precise, area-specific aggregation which our analyses confirmed as a non-normal, nonlinear pattern to the regional distribution (Fig. 1c; D’Agostino and Pearson normality test; K2 = 51.67, *p* < 0.0001, passed normality = no; nonlinear Regression; *y* = −354.1*ln(x) + 1200.9, *R*^2^ = 0.82465). The skewed data suggest that fewer brain regions have high burden, consistent with increasingly specific patterns of aggregation at the earliest stages of amyloidosis. To determine which regions have aggregates at 4 M, we looked at our clustering analyses (Fig. 1h) which revealed that regions homologous to the those in the DMN (e.g., retrosplenial (RSP), anterior cingulate (ACC), and parietal cortices)^34^ are better correlated to each other (Table 1) and first show Aβ in 4 M brains (Table 2). At 4 M, there was no appreciable Aβ in olfactory or extended limbic areas (Fig. 1h).

Human functional studies have suggested that the DMN is the earliest affected functional network^35,36^. Thus, to determine if our murine amyloid model reflected this human finding, we next looked at 2 M brains to understand whether there are earlier affected areas. At 2 M, there were very few brain regions with observable Aβ (Fig. 1f), and the brains failed to demonstrate significant differences across areas (Fig. 1c; D’Agostino and Pearson normality test; K2 = 105.2, *p* < 0.0001, passed normality = no; Friedman test; *Q* = 58.18, *p* = 0.1073). The unbiased clustering revealed that at 2 M, Aβ deposition is similar across most of the brain, confirming our density observations (Fig. 1h). However, the clustering analysis also indicated a few specific areas with distinct and correlated deposition in the 2 M animals (Table 2). These key nodes of early aggregation were the MB, SEPT, and SUB—regions that connect the HPC to the rest of the Papez memory circuit^37^. At 1 M, animals did not show discernable Aβ accumulation anywhere, except for a few small deposits in the MB (Fig. 1g), and these results are consistent with previous reports^28^ that deposition is largely not developmental. Overall, the spatiotemporal map supports a model where the SUB, MB, and SEPT are uniquely susceptible to Aβ aggregation and show the densest Aβ because they accumulate pathology over a longer period. This suggests that, over time, regional Aβ burden progresses to include more areas and that accumulation within a brain region may be dependent on factors that confer differential propensities to develop aggregates.

**Fig. 2:**
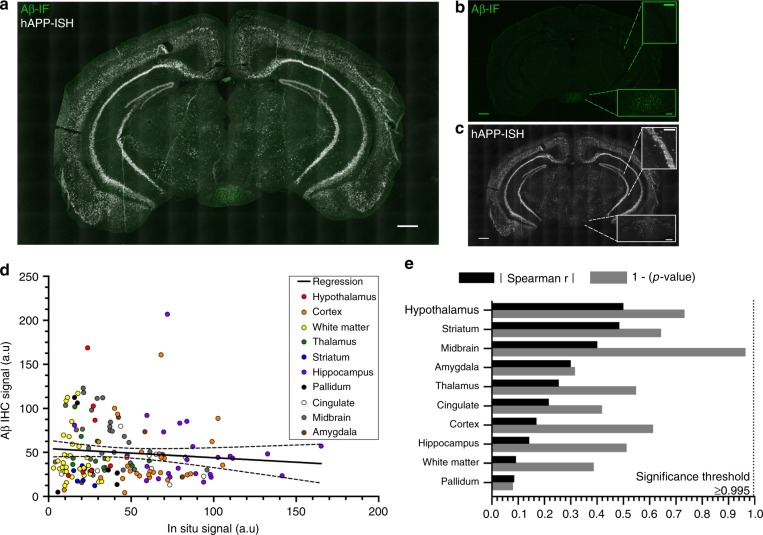
Progression patterns cannot be described by transgenic mRNA expression. **a** Representative merged image of amyloid immunofluorescent labeling (green) and APP mRNA in situ hybridization (white). Scale bar 500 μm. **b** Unmerged image of amyloid immunofluorescence signal. Scale bar 500 μm. Insets show mammillary body area with significant Aβ accumulation and hippocampus with very little Aβ accumulation. Inset scale bars 150 μm. **c** Unmerged image of APP in situ hybridization. Scale bar 500 μm. Mammillary body area shows lower hAPP mRNA expression than the hippocampus. Inset scale bars 150 μm. **d** Correlation between amyloid-IF signal and APP in situ. Colored dots represent separately quantified regions. Linear regression and correlation analyses carried out on the combined data. Linear regression: *Y* = −0.1027*x + 54.28; *F*_(1,157) _= 1.333, *p* = 0.2501. No significant deviation from zero. Spearman *r* = −0.07284; *p* = 0.3616. **e** Spearman correlation for in situ hybridization–immunofluorescence data by region. Black bars represent Spearman rho, gray bar represents 1−*p*-value of Spearman correlation. Significance threshold alpha = 0.05 with Bonferonni correction for ten regions

To ascertain whether the subcortical aggregation pattern is an artifact of the mouse transgene, we performed dual immunofluorescence for Aβ aggregates and in situ hybridization for the transgenic mRNA, so we could correlate the location of Aβ deposits with expression patterns of the transgene (Fig. 2a). We expected the transgene expression to remain stable across differently aged brains and, in our analyses, patterns of transgene expression in both 2 M and 4 M animals looked comparable across the subregions we analyzed. The correlation analyses between transgenic mRNA and Aβ deposits at the early time points demonstrates that across the entire brain, transgenic RNA expression does not correlate with deposit location, and many brain areas that have high transgene expression do not have plaques, even up to 4 M-old brain (Fig. 2b; Spearman correlation *r* = −0.0728, *p* = 0.3616). The results remained nonsignificant when we carried out the analysis splitting the data by brain region (Fig. 2c). The lack of correlation between Aβ aggregates and APP mRNA suggests that plaque burden in the 5XFAD animals is not exclusively dependent on the levels of transgene expression and that the brains show biologically relevant, region-specific amyloid deposition.

Together the spatiotemporal map in mice and biological relevance of region-specific deposition show that the core regions connecting the HPC to the rest of the Papez circuit—the MB, SEPT, and SUB—are particularly prone to Aβ deposition. Then, over time, the aggregates appear in increasingly complex cognitive systems moving next to the DMN, followed by the extended limbic system, before finally developing throughout the entire forebrain. Importantly, looking at human MB using SWITCH (Supplementary Movie 3; subject information in Supplementary Table 1) or immunofluorescent labeling (Supplementary Fig. 2A; subject information in Supplementary Table 2) demonstrates Aβ deposition is significantly higher in individuals with a clinical diagnosis of AD (Supplementary Fig. 2B, Shapiro–Wilk normality test, healthy: *W* = 0.7735, *p* = 0.0069, passed normality = no, AD: *W* = 0.9129, *p* = 0.4561, passed normality = yes; Kolmogorov–Smirnov test, *D* = 0.6667, *p* = 0.0420) and that higher percent MB area with Aβ correlated with increased likelihood of pathological diagnosis of AD (Supplementary Fig. 2C, Spearman correlation, CERAD: Spearman *r* = −0.4142, *p* = 0.1115, Braak: *r* = 0.5774, *p* = 0.0210, NIA-Reagan: *r* = −0.5086, *p* = 0.0459). This suggests that human brain alterations in AD look similar to what we observe in mouse. Thus we propose that Aβ deposits start in susceptible subcortical structures and spread to increasingly complex memory and cognitive networks with age.

### Papez structures are susceptible to dysfunction

**Fig. 3:**
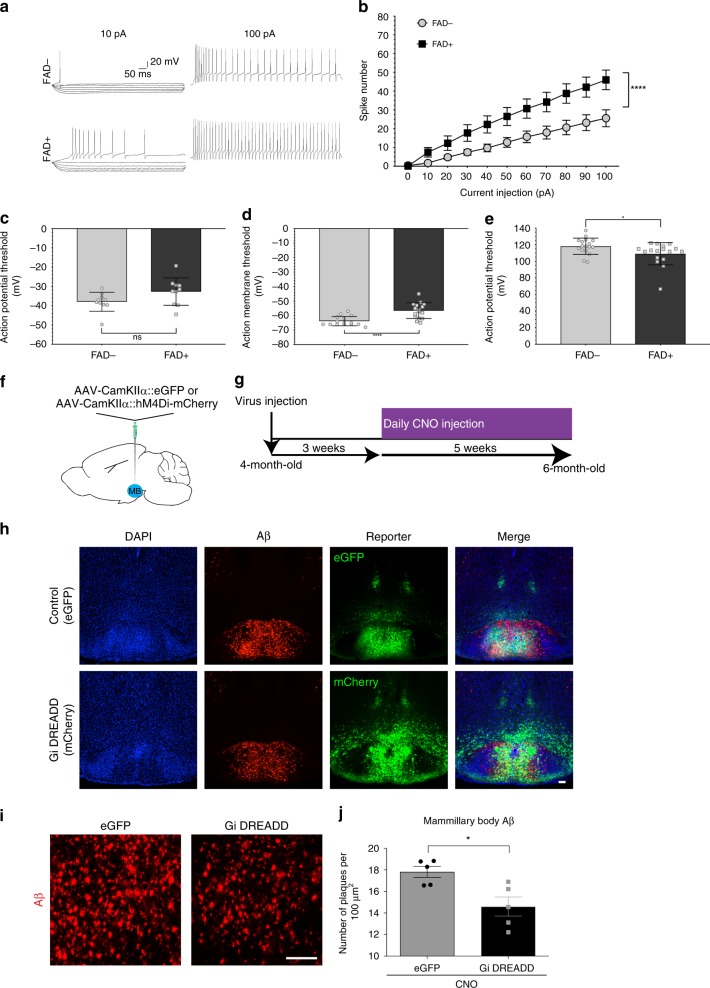
MB shows significant functional alterations in young 5XFAD mice. **a** Representative traces of neuronal patch-clamp recordings from mammillary body slices from 5XFAD + (FAD + ) and 5 × FAD- (FAD-) at 10 pA and 100 pA. **b** Excitability curve showing the spike number from 5XFAD + and 5XFAD− mammillary body neurons at each current injection step from 0 to 100 pA. Repeated measures ANOVA. Genotype: *F*_(1,31)_ = 7.166, *p* = 0.0118; current injection: *F*_(10,310)_ = 94.22, *p* < 0.0001; interaction: *F*_(10,310) _= 6.855, *p* < 0.0001. *N* = 17 cells/group. Graph reports mean ± standard error. **c** Action potential threshold in mammillary body neurons is not significantly different between 5XFAD + and 5XFAD−. Unpaired Student’s *t* test; *t*_(18)_ = 1.924, *p* = 0.0704. Each dot represents a single cell. Graph reports group mean ± standard deviation. **d** Resting membrane potential is significantly depolarized in 5XFAD mammillary body neurons compared with 5XFAD−. Difference between means = 7.24 mV. Unpaired Student’s *t* test; *t*_(29)_ = 4.509, *p* < 0.0001. Each dot represents a single cell. Graph reports group mean ± standard deviation. **e** Action potential amplitude is significantly decreased in 5XFAD + mammillary body neurons compared with 5XFAD−. D’Agostino and Pearson normality test, FAD-: K2 = 0.6008, *p* = 0.7405, passed normality = yes; FAD + : K2 = 20.61, *p* < 0.0001, passed normality = no; Mann–Whitney *U*; *U* = 77, *p* = 0.0196. Each dot represents a single cell. Graph reports group mean ± standard deviation. **f** Cartoon illustrating the injection of AAV-CamKIIα::eGFP or AAV-CamKIIα::hM4Di-mCherry (Gi DREADD). **g** Diagram illustrating the time course of virus and CNO injections in the 5XFAD mice. **h** Images of the mammillary body in AAV-CamKIIα-EGFP or AAV-CamKIIα-hM4Di-mCherry (Gi DREADD) injected 5XFAD mice. DAPI (blue), Aβ (red), and reporter (green: EGFP or mCherry. Scale bar 100 μm. **i**–**J** Images (**i**) and quantification (**j**) of Aβ plaque (red) in the mammillary body of control and Gi DREADD mice. Scale bar 100 μm. Unpaired Student’s *t* test; *t*_(8)_ = 3.137, *p* = 0.0139. *p < 0.05. *N* = 5 mice per group. Graph reports mean ± standard error

A prevailing hypothesis is that Aβ-induced electrophysiological dysfunction underlies cognitive decline and studies have shown that hyperexcitability—like that seen in AD patients—can increase the deposition of Aβ. Thus, we set out to determine whether alterations in neuronal activity like those in the cortex and hippocampus may also be a factor that contributes to regional vulnerability in the subcortical structures we identified^7,25,38,39^. In our spatiotemporal map data, we found that the MB showed the densest Aβ at the earliest stages. This region plays a key role in spatial and anterograde memory in mice and humans^40–42^, and these two aspects of cognitive function decline in AD. Thus, we wanted to determine whether the Aβ accumulation we observed in the MB was associated with any functional changes that may confer susceptibility. We performed ex vivo whole-cell patch-clamp recordings in MB in slices from 5XFAD mice and littermate controls. Even at 2 M, we found that 5XFAD MB neurons showed significantly higher intrinsic excitability (Fig. 3a), with a significantly larger number of action potentials elicited by incremental depolarizing current steps (Fig. 3b, two-way repeated measures ANOVA; interaction: *F*_(10,310)_ = 6.855, *p* < 0.0001; genotype: *F*_(1,31)_ = 7.166, *p* = 0.0118). Although the threshold for action potentials in MB neurons did not differ between groups (Fig. 3c, unpaired Student’s *t* test; *t*_(18)_ = 1.924, *p* = 0.0704), the resting membrane potential differed significantly from WT controls and was on average ~7.3 mV more depolarized in MB neurons from 2 M 5XFAD mice (Fig. 3d; unpaired Student’s *t* test; *t*_(29)_ = 4.509, *p* < 0.0001). Finally, we also observed a slightly increased action potential amplitude in 2 M 5XFAD mice (Fig. 3e; D’Agostino and Pearson normality test, FAD-: K2 = 0.6008, *p* = 0.7405, passed normality = yes; FAD + : K2 = 20.61, *p* < 0.0001, passed normality = no; Mann–Whitney *U*; *U* = 77, *p* = 0.0196), which likely also contributes to the overall observed hyperexcitability. This observation suggests that high levels of Aβ contribute to dysfunction, even in deep, subcortical structures.

### Modulating activity in the MB reduces Aβ pathology

Studies have reported that neuronal activity can regulate Aβ production and deposition such that inducing neuronal activity increases Aβ levels and inhibiting activity reduces it^43–46^. To test whether the hyperexcitability we observed in the MB affects Aβ deposition in 5XFAD mice, we modulated neuronal activity in MB excitatory neurons using an inhibitory Designer Receptor Exclusively Activated by Designer Drug (DREADD)^47^. We stereotaxically injected either AAV-CamKIIα-eGFP (eGFP) or AAV-CamKIIα-hM4Di-mCherry (Gi DREADD) into MB of 4-month-old 5XFAD mice (Fig. 3f). We allowed for 3 weeks of postoperative recovery and time for expression of viral vectors and then gave daily intraperitoneal (IP) injections of clozapine-N-oxide (CNO) to activate the inhibitory DREADD receptors to reduce MB activity over a period of 5 weeks (Fig. 3g). We confirmed the expression of eGFP or hM4Di-mCherry in MB (Fig. 3h) and analyzed whether prolonged reduction of MB activity affected the amount of Aβ in the region (Fig. 3i). We saw a significant reduction in MB Aβ in the Gi DREADD-expressing group compared with eGFP controls (Fig. 3j, Unpaired Student’s *t* test; *t*_(8)_ = 3.137, *p* = 0.0139. *p *<* 0.05). Because regulation of MB activity affected Aβ deposition, this finding indicates that the hyperexcitability we observed in MB neurons may contribute to the worsening Aβ load in the region over time. In addition, because we intervened at an advanced stage of amyloidosis, our data also suggest that regulating activity can slow pathological progression later in the disease.

To understand whether chronically inhibiting activity in the MB also led to changes of Aβ deposition in the interconnected brain regions, we further measured the amount of Aβ plaque in the dorsal subiculum, an upstream brain region that project to the MB, and the anterior thalamus, a brain region downstream from the MB^42^. We found that prolonged inhibition of the MB does not significantly alter Aβ in the dorsal subiculum (dSUB) (Supplementary Fig. 3A, unpaired Student’s *t* test; *t*_(8)_ = 1.173, *p* = 0.2745), suggesting that manipulating activity does not affect Aβ deposition in the upstream brain region. We then tried to quantify Aβ deposition in the anterior thalamus downstream of the MB. In doing so, we observed too little Aβ to compare between the control and Gi DREADD groups (Supplementary Fig. 3B). Although the anterior thalamus is the primary output of the MB, we also noticed eGFP- and mCherry-labeled axons in the dentate gyrus (DG) (Supplementary Fig. 3C). Upon further investigation, we observed expression of eGFP and Gi DREADD in the supramammillary body (SUM), a brain region immediately adjacent to the top of the MB (Fig. 3h) that projects to the DG. Because we observed this in the majority of animals we used in the studies, we concluded that our injection technique did not allow us to restrict AAV to the MB without also introducting virus into the SUM.

We decided to use the presence of eGFP or Gi DREADD in the SUM to understand how manipulating activity affects Aβ in downstream regions, because we were unable to do so from the MB. To do this, we analyzed Aβ deposition in the DG following our prolonged inhibition paradigm. We observed a reduction of Aβ plaque in the DG after prolonged inhibition of activity in the SUM (Supplementary Fig. 3C, unpaired Student’s *t* test; *t*_(8)_ = 2.653, *p* = 0.0291. *p < 0.05). Although little Aβ plaque is observed in the SUM at this age, this result importantly indicates that chronically inhibiting activity in one brain region can lead to a reduction of Aβ plaque in the downstream brain region.

## Discussion

Using optimized SWITCH whole-brain clearing and immunolabeling technologies, we created a spatially unbiased map of the progression of Aβ deposits that revealed area-specific aggregation over time and revealed novel subcortical hubs of early-disease susceptibility in the 5XFAD mouse model of AD. We showed that the initial accumulation of Aβ correlates with electrophysiological changes, which then confer additional susceptibility to accumulating pathology. Our data suggest that subcortical memory network hubs may be critically susceptible to pathological changes that occur in AD and that alterations within them may contribute to memory loss in Alzheimer’s dementia.

Papez circuit dysfunction is a prevailing theory of memory loss in AD. Although the MB is a critical part of the Papez long-term memory circuit connecting the hippocampus to the anterior thalamus^37^, it has not been strongly implicated in Alzheimer’s dementia^37,48–51^. This is somewhat surprising because its major inputs, the subiculum and prefrontal cortex^52^, have been shown to demonstrate substantial synaptic loss that correlates with memory performance^53–56^. In addition, the septum, which is another core node in the Papez circuit, was initially implicated in early AD by the cholinergic hypothesis. This idea posited that loss of neurons in the septum and other cholinergic nuclei were causative in disease onset and progression^57^. Although the idea was overshadowed by the amyloid cascade hypothesis^2^, integrating our findings with the data on the hippocampus, subiculum, and septum, there is now strong evidence that each of the nodes in the Papez circuit is particularly vulnerable early in AD. Our data, along with other recent evidence for Papez circuit dysfunction^58,59^, suggest that not only do these subcortical structures develop Aβ deposits early in AD but that they also demonstrate neural activity changes, cellular and synaptic loss, and pathological tau aggregation that may contribute to circuit-wide deficits that likely affect cognition^60^. Although previous studies have focused on different types of Aβ or shown correlational relationships between regions, the data in this paper strengthen the evidence for dysfunctional memory circuits as a core pathology underlying AD both by revealing Papez-wide susceptibility to Aβ deposition using novel techniques and by demonstrating functional consequences and pathological implications of Aβ in an underexplored node in the circuit^7,25,39,61^.

In addition to enhancing our understanding of the regions and circuits that show primary Aβ aggregation and that undergo early functional destabilization, our data suggest Aβ propagates between connected brain regions by showing the affliction of increasingly complex yet interconnected memory networks with age^62^. This provides a unifying view of early lesions that were previously at odds between pathological and neuroimaging data, creating a framework in which to better interpret human pathogenesis. Investigators have suggested that the DMN, limbic system, attentional systems, and brain stem may all be involved at the earliest stages of prodromal AD. The data presented in this paper suggest that these do not need to be disparate hypotheses, but instead represent different stages of network affliction that occur as the disease progresses. In the mouse model, 2 M 5XFAD animals show core limbic affliction, followed by the DMN at 4 M and typical limbic system at 6 M. These observations suggest that early AD may be best staged by the network aberrations detected by functional MRI techniques. These alterations occur before overt memory loss^36^, and with increasing evidence for restoration of network function as a successful treatment in AD models^7,63,64^, our data may lay the ground work for network-progression staging to guide early-disease circuit interventions.

In humans with Alzheimer’s dementia, a slow accumulation of altered cellular processes, including Aβ deposition, and disrupted neural circuits long precedes cognitive deficits, such that clinical diagnosis often does not happen until much later in the progression of AD^27,65^. In this study, we demonstrate a similar propensity for early Aβ accumulation and altered electrophysiological function as early as 2 M in the 5XFAD model, with a delay in behavioral impairments until ~6 M of age. We utilized a chemogenetic approach to reduce MB activity in this key node of the Papez circuit, and found a significant reduction in MB Aβ levels. Importantly, we did not manipulate activity levels until animals were almost 5-month-old, when Aβ pathology has already spread to additional brain regions and networks. This suggests that interventions within key nodes may have the potential to impact pathology, and that it may be possible to alter cognitive outcomes with this type of strategy even near the projected onset of cognitive decline. Because the regions in the basal forebrain are linked in our data, and other studies, to the hypothalamic diencephalic limbic structures, we suggest in-depth examinations of these regions and their connections may lead to a better understanding of the mechanisms of dysfunction underlying AD onset and the ensuing progressive memory loss.

## Methods

### Animals

All mouse work was approved by the Committee for Animal Care of the Division of Comparative Medicine at the Massachusetts Institute of Technology. 5XFAD (Tg 6799) breeding pairs were acquired from the Mutant Mouse Resource and Research Center (MMRRC) (Stock No. 034848-JAX) and maintained as hemizygous on the BL6 background. Animals were group housed on a 12 h light/dark cycle with Nestlet enrichment and killed at ages 18 M, 12 M, 6 M, 4 M, 2 M, 1 M as noted in the text.

### Mouse tissue fixation

Mice were deeply anesthetized with isoflurane (Isoflurane, USP, Piramal Healthcare, Andhra Pradesh, India) and underwent transcardial perfusion with ice-cold 1× PBS (10× stock, Gibco, #70011–044) followed by ice-cold fixative made of 4% paraformaldehyde (32% stock, Electron Microscopy Sciences (EMS), Hatfield, PA, #15714) and 1% glutaraldehyde (10% stock, EMS #16110) in 1× PBS. Brains were removed from the skull and postfixed in the same fixative for 3 days shaking at 4 °C.

### Intact mouse brain SWITCH processing

After washing in 1× PBS, brains incubated in inactivation solution of 1% acrylamide (40% stock, Biorad #161–0140), 1 M glycine (Sigma-Aldrich, St. Louis, MO, #G7126) in 1× PBS. After washing in 1× PBS, brains were put into clearing solution of 200 mM sodium dodecyl sulfate (SDS) (Sigma-Aldrich, #L3771), 20 mM lithium hydroxide monohydrate (Sigma, #254274), 40 mM boric acid (Sigma-Aldrich, #7901), pH 8.5–9.0 and left shaking at 55 °C for 4 weeks until white matter tracts were translucent to the eye in SDS. Brains were washed in 1× PBS or Weak Binding Solution (WBS) for up to 1 week and immunolabeled with the SWITCH protocol for labeling intact mouse brain (Supplementary Methods).

### Mouse brain section processing

Where sections were analyzed, brains were sliced to 100 µm on a vibratome (Leica VT100S) and stored at 4 °C in 1× PBS + 0.02% sodium azide (Sigma-ALdrich, #S2002). For SWITCH labeling, individual sections were incubated in clearing solution shaking at 55 °C for 2 h and were washed in 1× PBS. Sections were immunolabeled with the SWITCH protocol for labeling mouse brain sections (Supplementary Methods), and antigens targeted are noted in the text.

### Intact human tissue SWITCH processing

Human tissue blocks were deparaffinized by sequential immersion in xylene, ethanol, and water (details in Human SWITCH protocol). Then, blocks were incubated in 1% GA in 1× PBS for 10 days at 4 °C. Brains were incubated in clearing solution shaking at 55 °C until the tissue appeared translucent (4–8 weeks). Following clearing, tissue was labeled using the SWITCH protocol for labeling human autopsy specimens (Supplementary Methods).

### Human specimen tissue section processing

Tissue from the Netherlands Brain Bank (Supplementary Movie 3): Formalin-fixed paraffin-embedded human postmortem tissue blocks were sectioned at 5-µm thickness, dried at room temperature for 24 h and heated at 80 °C for 24 h before IHC processing. Deparaffinization, antigen retrieval, and subsequent staining were performed with Boston Medical Center Pathology Department’s Ventana Benchmark Ultra automated IHC instrument using commercially available primary antibodies specific for Aβ (mouse anti-human beta-amyloid [6 F/3D] monoclonal antibody, 1:50, Dako, Glostrup, Denmark), visualized by HRP-conjugated secondary antibody with diaminobenzidine (DAB) chromogen.

Tissue from the Religious Orders Study (Supplementary Fig. 2): Formalin-fixed tissue was embedded in 2% agarose gel and sectioned at 40 -µm thickness into 1× PBS. Slices were blocked with 2% bovine serum albumin in 1× PBS with 0.1% triton x-100 and then incubated in CST-D54D2 anti- Aβ primary. After washing, sections were incubated in AlexaFluor-conjugated secondary. After a final wash, including DAPI for nuclear identification, sections were mounted for imaging in Fluoromount G.

### Antibodies and dyes

The primary antibodies used are shown in Table 3. Hoechst 33528 (Sigma #14530) and DAPI (ThermoFisher Scientific, #D1306) were used for nuclear labeling. All secondary antibodies were Pre-adsorbed F(ab)2’ AlexaFluor-conjugated from AbCam.

**Table 3:** Primary antibodies used in this study

Target	Host	Company	Product #	Dilution
Aβ [D54D2]	Rabbit	Cell Signaling Technologies	8243	1:100–1000
Myelin Basic Protein^a^ SMI-99	Mouse	BioLegend	808401	1:50–500
Myelin Basic Protein^a^ SMI-94	Mouse	BioLegend	836502	1:50–500
β-amyloid [6 F/3D]	Mouse	Dako	M087201–2	1:50

^a^These antibodies were used concurrently as per the manufacturer’s recommendation

### Intact brain image acquisition

Intact brain images were acquired on a custom SPIM microscope built by H.C. During imaging, samples were illuminated with a sheet of light generated by scanning a focused beam from a light source (SOLE −6 with 488, 561, 647 nm, Omicron) through a low NA objective (Macro 4X/0.28 NA, Olympus) with a galvo-scanner (6215 H, Cambridge Technology). Collection of emitted light on the microscope occurs through a long working distance high NA objective (10x/0.6 NA WD 8 mm CLARITY, Olympus). The microscope is outfitted with four sCMOS cameras (Orca Flash4.0 V2, Hamamatsu) for simultaneous multichannel signal recording. During acquisition, the samples were illuminated simultaneously from dual illumination arms (one on each side) to minimize the shading effects of light-scattering elements in the brain. Dynamic confocal mode of detection is implemented by synchronizing the scanning speed of the illumination beam with the read-out speed of the rolling shutter mode of sCMOS camera, which improves the signal to background ratio by filtering out background signal from out-of-focal regions. Sample is mounted on a motorized stage with *x*, *y*, *z* translation and theta rotation (two of M-112.2DG, M-111K028, M-116.DG, Physik Instrumente) for mosaic imaging. Z-stack imaging by sample scanning alone is slow due to communication overhead between the host computer and the stage controller. We achieved high-speed volume imaging by scanning the light sheet along the depth direction with a galvo-scanner and synchronizing the position of the light sheet with the detection objective’s focal plane by moving the detection objective with a piezo actuator (P-628.1CL, Physik Instrumente). To maintain light sheet position on the focal plane of the objective across the entire sample volume, we implemented an image-based autofocusing algorithm^66^. The laser settings are determined such that ~5% of the images are saturated to its maximum gray level for high signal to background ratio. The sample chamber is filled with the refractive index-matching solution (RIMS)^67^. Depending on the refractive index of the medium, the beam waist position of the illumination light sheet shifts along the illumination beam direction. Each illumination objective is mounted on the piezo actuator (P-628.1CL, Physik Instrumente) to allow the beam waist position to be adjusted to the center of the detection objective. Sample chamber is specially designed to allow for free motion of the detection objective while preventing leakage of the immersion medium.

### Intact brain image processing

Each tile is first corrected for the nonuniform illumination pattern using a modified algorithm from Smith et al.^68^. Multiple stacks of acquired images are stitched with Terastitcher^69^. Each tile has 15% overlapping area with the neighboring tiles for calculating stitching parameters. The voxel size of raw data is 0.58 × 0.58 × 5.0 µm. The raw data set is first down-sampled four times in *X* and *Y* dimension and then stitched to ease the computation burden in the downstream analysis. The stitched data set is analyzed with Imaris software (Bitplane).

### Section image acquisition

For section and human tissue imaging, tissue was mounted onto microscope slides (VWR VistaVision, VWR International, LLC, Radnor, PA, USA) with either Fluromount G Mounting Medium (Electron Microscopy Sciences, Hatfield, PA, USA) or RIMS solution^67^.

Confocal slice images were acquired on a Zeiss LSM Inverted 710 microscope using Zen 2012 software (Carl Zeiss Microscopy, Jena, Germany). Images with cellular resolution were taken using a C-apochromat 40X, water immersion objective, NA 1.20. Section overview images used a Plan-apochromat 5X, air objective, NA 0.16. Pinhole, optical sectioning, and laser settings were determined for each experiment, and kept consistent for all images within an experiment or that were included within one analysis.

### Human brain image acquisition

Human brain images were acquired on a Leica TCS SP8 Confocal Microscope using LASAF software (Leica Microsystems, Wetzlar, Germany). Images were taken using a ×25 1.0NA CLARITY optimized objective with 6 mm working distance. The pinhole, optical sectioning, and resolution and laser settings were empirically determined for one brain, and kept constant for imaging subsequent samples.

### 3D image quantification

Images were analyzed using Imaris (Bitplane, Zurich, Switzerland). All quantification steps were performed on raw images by blinded investigators. For intact tissue analyses (Fig. 1), each brain file was segmented by hand using white matter tract and regional guidelines from the Allen Brain Atlas (Allen Mouse Brain Atlas, Coronal) to delineate boundaries for each major brain region (Supplementary Movie 4). After segmentation, a spots object was created on a 12-month brain. The parameters were fixed over the entire brain, and spots were separated into the bounded brain regions using the Spots into Surfaces tool in Imaris Xtensions. A spots object was created on each brain, and these objects were similarly split into brain regions using the Xtension. The data were exported to CSV and analyzed GraphPad Prism 7.0a for Mac.

### 2D image quantification

For 2D analyses, images were imported into FIJI^70^ as LSM files. Numerical data were saved in a spreadsheet and exported to GraphPad Prism 7.0a for Mac for statistical analyses. For ISH-IF analyses, blinded observers outlined three regions of interest (ROI) within each brain area looking only at the image containing APP-ISH information. The same regions were overlaid on the amyloid-IF image for quantification of the Aβ signal. For human amyloid analyses (Supplementary Fig. 2), two blinded observers counted plaques within images using the Multi-point tool. Then the segmentation was overlaid on the Aβ channel as a selection, within which a threshold was applied to the images. Finally, a Analyze Particles tool was used to count individual deposits. In all figures with 2D images, quantification was performed on raw, unaltered image data. For visibility at print resolution, representative images in Fig. 2 have had the brightness and contrast adjusted. All contrast and brightness adjustments were made identically across all images within a figure. Raw images are available upon request.

### Representative images and supplementary movies from intact brain data

Representative images from the intact brain data sets are either 2D images of the 3D-rendered data set or digitally sectioned at 5–100 µm in Imaris using the Orthoslicer tool. Supplementary Movies are created using the Key Frame Animation tool in Imaris. The brightness of the images has been individually adjusted for each brain to enhance 2D/3D viewing of specific objects. Because of these alterations, no direct comparisons of labeling intensity should be made between images.

### In situ hybridization probe design

RNA antisense probes were generated by PCR-amplifying human cDNA with human-specific *APP* primers with a T7 RNA polymerase recognition sequence (TAATACGACTCACTATAGGG) fused to the reverse primer (Table 4). The resulting PCR product was gel extracted and in vitro transcribed using a DIG-RNA labeling kit (Roche).

**Table 4:** Primer sequences (5′−3′) for in situ probe preparation

Gene	Forward primer	Reverse primer
h*APP*	GAGACACCTGGGGATGAGAA	TAATACGACTCACTATAGGGACAGAGTCAGCCCCAAAAGA

### Immuno-in situ hybridization (Immuno-ISH)

Mice were anesthetized by isoflurane in an open system and perfused with RNase-free PBS followed by RNase-free 4% formaldehyde. Brains were dissected, drop fixed in RNase-free 4% formaldehyde for 12 h, equilibrated in 30% sucrose-PBS, and frozen in O.C.T. (TissueTek). Cryosections (10 μm) were incubated with a DIG-labeled RNA antisense probe (1:1000 in hybridization buffer) overnight at 65 ^o^C, washed in 1× SSC/50% formamide/0.1% Tween-20 3× 30 min at 65 ^o^C followed by 1× MABT for 30 min at room temperature. Sections were blocked with 20% heat-inactivated sheep serum/2% blocking reagent (Roche)/1× MABT for 1 h and then incubated with mouse anti-DIG antibody (Roche; 1:2000) and rabbit anti-amyloid β (Cell Signaling; 1:500) diluted in blocking solution overnight. Sections were washed with 1× MABT 2× 20 min, incubated with donkey anti-rabbit Alexa-488 (Invitrogen; 1:1000) diluted in blocking solution for 1 h, and washed with 1× MABT 5× 20 min. Sections were then prestained with 100 mM NaCl/50 mM MgCl_2_/100 mM Tris pH 9.5/0.1% Tween-20 2× 10 min, followed by staining with NBT/BCIP (Roche; 4.5 μl/ml and 3.5 μl/ml, respectively, in prestaining buffer) for 2 h. Sections were washed with 1× PBS 3× 15 min, incubated in xylene 3× 5 min, and mounted with VectaMount (Vector Laboratories).

### Slice electrophysiology

Acute brain slices were prepared from male and female 5XFAD mice and WT littermate controls, aged 2–2.5-months old. The experimenter was blinded to the group of animal. The mice were anesthetized with isoflurane and decapitated. After decapitation, the brains were rapidly removed, and a cut was made to remove the cerebellum. The brain was mounted anterior-side up. Coronal brain slices (250 -μm thick) were prepared in ice-cold dissection buffer bubbled with 95% O_2_–5% CO_2_ containing (in mM) 211 sucrose, 3.3 KCl, 1.3 NaH_2_PO_4_, 0.5 CaCl_2_, 10 MgCl_2_, 26 NaHCO_3_ and 11 D-glucose using a Leica VT1000S vibratome (Leica). Slices were recovered in a holding chamber with 95% O_2_/5% CO_2_-saturated artificial cerebrospinal fluid (ACSF) consisting of (in mM) 124 NaCl, 3.3 KCl, 1.3 NaH_2_PO_4_, 2.5 CaCl_2_, 1.5 MgCl_2_, 26 NaHCO_3_, and 11 D-glucose for 1 h at 32 °C and then stored at room temperature. Individual slices for recording were then transferred to a submerged recording chamber and perfused with ACSF at a constant rate of 2–2.5 ml/min at room temperature. Cells were visualized using infrared differential interference contrast (IR-DIC) imaging on an Olympus BX-50WI microscope. Action potentials (APs) in whole-cell current patch clamp from mammillary body were acquired on an EPC10 amplifier (HEKA Elektronik) with Patchmaster software. APs was elicited by current clamp of current steps from 0 pA to + 100 pA at 10 pA increments for 800 ms. Signals were filtered at 2 KHz and stored on a personal computer (PC). A borosilicate glass electrode (resistance of 6–7 MΩ) with pipette solution containing (in mM) 130 K gluconate; 20 KCl; 10 HEPES; 0.2 EGTA, 4 MgATP, 0.3 Na_2_GTP, 10 disodium phosphocreatine was used. APs and resting membrane potentials (RMP) were analyzed using Patchmaster software (HEKA Electronik). Statistics were calculated in Prism as described below and in the text. The data are represented as outlined in the text and figure legends.

### DREADD experiments

5XFAD mice were anaesthetized with isoflurane in the stereotaxic frame for the entire surgery, and their body temperature was maintained with a heating pad. In order to inhibit neuronal activity in the mammillary body (MB), 200 nl of adendo-associated virus carrying inhibitory DREADD, hM4Di and mCherry (pAAV-CamKIIα-hM4Di-mCherry, Addgene, catalog #50477-AAV8) were injected into the MB (From bregma: A|P: −2.0 mm, M|L: 0.0 mm, D|V: −5.6 mm). Injections were performed at a rate of 50 nl per min. The needle was allowed to sit in the target location for 3 min prior to the start of viral infusion and for 5 min after injection was completed. 5XFAD mice injected with AAV-CamKIIα-EGFP (AAV-CamKIIa-GFP, University of North Carolina Vector Core) in the MB were used as control group. We allowed for 3 weeks of postoperative recovery and time for expression of viral vectors. Mice were then given i.p. injections of clozapine-n-oxide (CNO, Tocris, catalog #4936/50) at 3 mg/kg body weight, daily for 5 weeks. CNO was dissolved in saline with a working solution at 0.5 mg/ml. To control the CNO effect, both control and mice with expression of inhibitory DREADD were injected with CNO in the experiment.

### Statistics and reproducibility

All statistics were performed in MatLab or GraphPad Prism. Individual statistical tests are indicated in the text and/or figure legends for the appropriate experiments. Data sets were checked for normality and statistics were run depending on the appropriateness of parametric or nonparametric tests for the normality of the data. Graphs were created in the respective analytical software packages and exported as .TIFF for inclusion in the document. Sample sizes reported in the text and figures represent numbers of independent samples used in the analysis (e.g., animal numbers, unique human patients, etc), unless otherwise noted in the text.

### Reporting summary

Further information on research design is available in the Nature Research Reporting Summary linked to this article.

## Supplementary information

Supplementary Information

Description of Additional Supplementary Items

Reporting Summary

Supplementary Movie 1

Supplementary Movie 2

Supplementary Movie 3

Supplementary Movie 4

## Data Availability

All data reported in this paper including raw image files are available upon request. Images are stored as.lsm,.czi,.tif or.lei files. Imaris files are stored as.ims. The data can be requested from the Tsai Laboratory by emailing tsaiasst@mit.edu or contacting one of the corresponding authors. Resources that may help enable general users to establish the methodology are freely available online (http://www.chunglabresources.org).
