# Supplementary material for: 3D mapping reveals network-specific amyloid progression and subcortical susceptibility in mice
Source: Commun Biol. 2019 Oct 4;2:360. doi: 10.1038/s42003-019-0599-8 (PMC6778135; doi:10.1038/s42003-019-0599-8)
Supplement: Supplementary file 1 — Supplementary Information [file 42003_2019_599_MOESM1_ESM.pdf]

Supplementary Figure 1

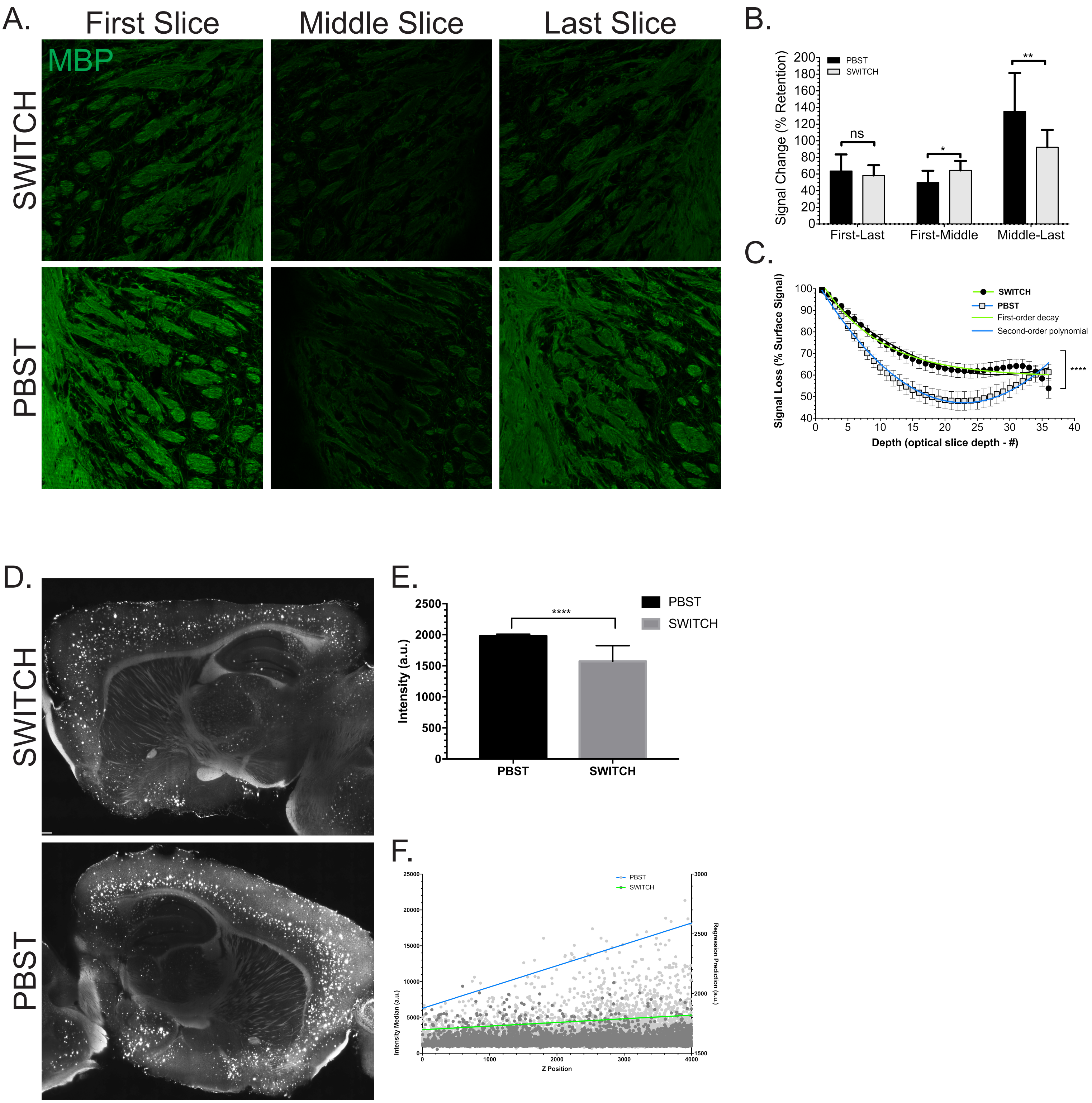

Supplementary Figure 1: SWITCH enables homogenous thick tissue labeling

A) Representative optical sections from the first (left), middle (center), and last (right) slices of z-stack image files taken from 100um thick tissue labeled for myelin basic protein (MBP, green) using either SWITCH buffers (top) or traditional labeling buffer of phosphate buffered saline with triton x-100 (PBST, bottom).

B) Quantification of intensity change between sections displayed in S1A displayed as percent signal retention. Significant increase in signal retention between the first and middle slices and better consistency between middle and last slices indicates more homogenous section labeling. N = 12/group. Multiple t-tests. No assumption of equal variance. First-Last: $t_{(22)} = 0.7988$, $p = 0.4329$; First-Middle: $t_{(22)} = 2.793$ $p = 0.0106$; Middle-Last: $t_{(22)} = 2.93$ $p = 0.0078$. Graph reports mean ± standard deviation.

C) Signal attenuation profiles for PBST and SWITCH processed samples. Non-linear regression. Comparison of fits: One-phase decay vs. Second order polynomial. SWITCH preferred model: One-phase decay; PBST preferred model: Second order polynomial. Global fit test (one-phase decay): $F_{(3,858)} = 55.1$, $p < 0.0001$. Different curve for each data set. Global fit test (second order polynomial): $F_{(3,858)} = 68.49$, $p < 0.0001$. Different curve for each data set. Graph reports mean ± standard error.

D) 12M aged 5XFAD brain was split into two hemispheres and labeled with either SWITCH buffers or PBST. Matched hemipheres were labeled for amyloid using CST-D54D2 SWITCH (top) or PBST (bottom).

E) Average intensity of amyloid labeling from representative optical sections. N = 2 hemispheres/group, 5 slices/hemisphere. Graph reports mean ± standard deviation.

F) Representative intensity signal plot throughout hemisphere. Intensity of spots in from SWITCH (dark gray) and PBST (light gray) matched hemisphere scattered on left y-axis. Linear regression PBST: $Y = 0.1785*X + 1876$; Linear regression SWITCH: $Y = 0.03058*X + 1697$. Comparison of fits: $F_{(2,13994)} = 101.2$, $p < 0.0001$.

Supplementary Figure 2

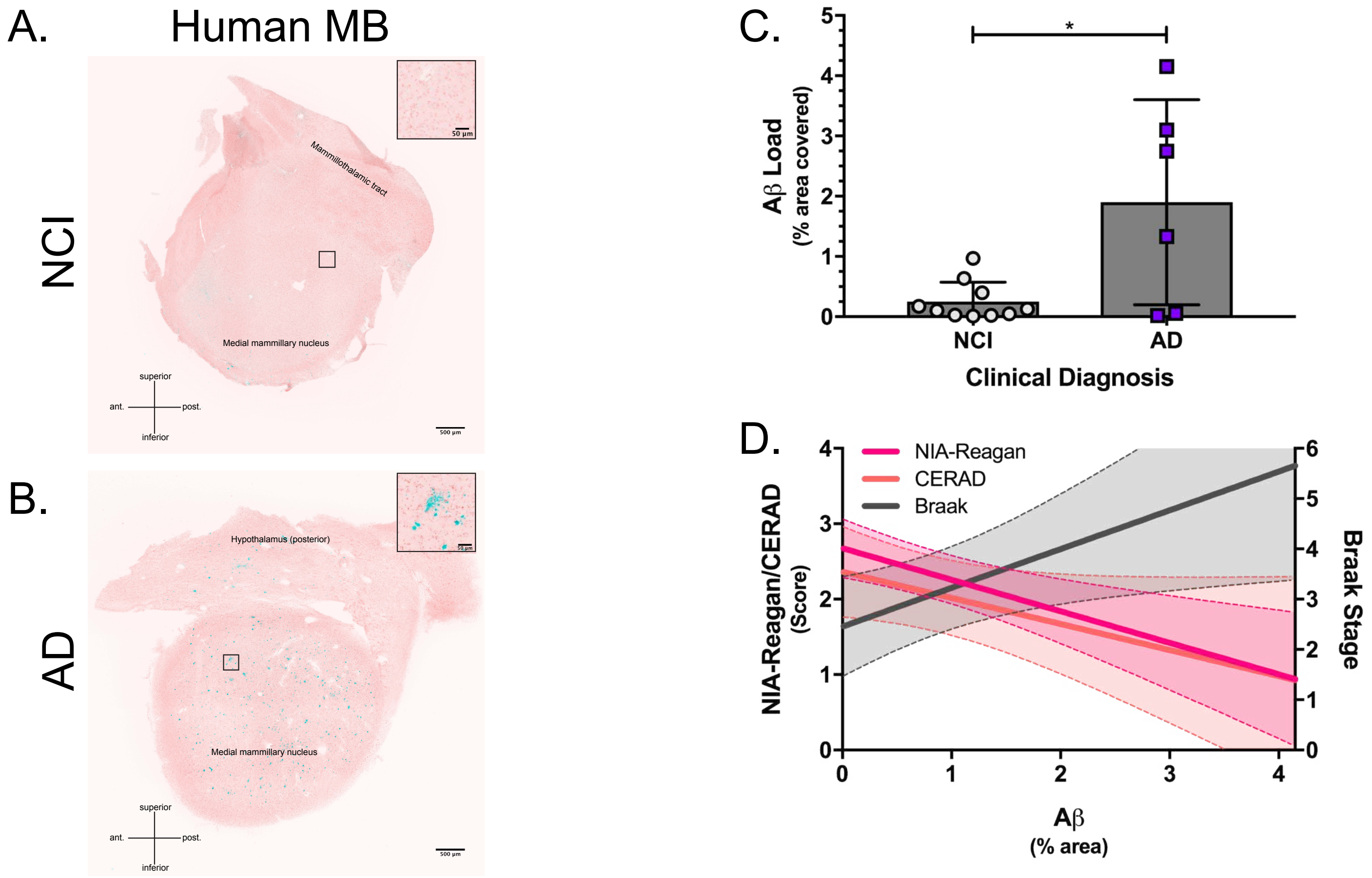

Supplementary Figure 2: Human mammillary body shows AD-related amyloid burden

A) – B) Representative images of 40um sections from human MB from the Religious Orders Study.[1] Individuals with no cognitive impairment (NCI) showed few deposits (A), while individuals with a clinical diagnosis of AD (AD) had significant plaque accumulation (B). Aβ = amyloid beta, cyan; DAPI – red.

C) Quantification of plaque load defined by percent area covered demonstrates significantly more Aβ in MB from individuals cognitively diagnosed with AD (AD) than those with no cognitive impairment (NCI). Shapiro-Wilk normality test, Healthy: $W = .7735$, $p = 0.0069$, passed normality = no, AD: $W = 0.9129$, $p = 0.4561$, passed normality = yes; Kolmogorov-Smirnov test, $D = 0.6667$, $p = 0.0420$. Graph reports mean ± standard deviation.

D) Mammillary body Aβ significantly correlates with Braak stage and NIA-Reagan scores and trends toward correlation with CERAD. Spearman correlation, CERAD: Spearman $r = -0.4142$, $p = 0.1115$, Braak: $r = 0.5774$, $p = 0.0210$, NIA-Reagan: $r = -0.5086$, $p = 0.0459$ NIA-Reagan/CERAD plotted on left y-axis (Likelihood of AD: 1 = AD diagnosis, 4 = No AD); Braak stage plotted on right y-axis (Presence of NFT: 0 = none, 6 = NFT seen in all areas observed). Linear regression, CERAD: $Y = -0.3457*x+2.363$, Braak: $Y = 0.7707*x + 2.455$. NIA-Reagan: $Y = -0.4181*x + 2.676$. Graph reports linear regression ± 95% confidence interval

Supplementary Figure 3

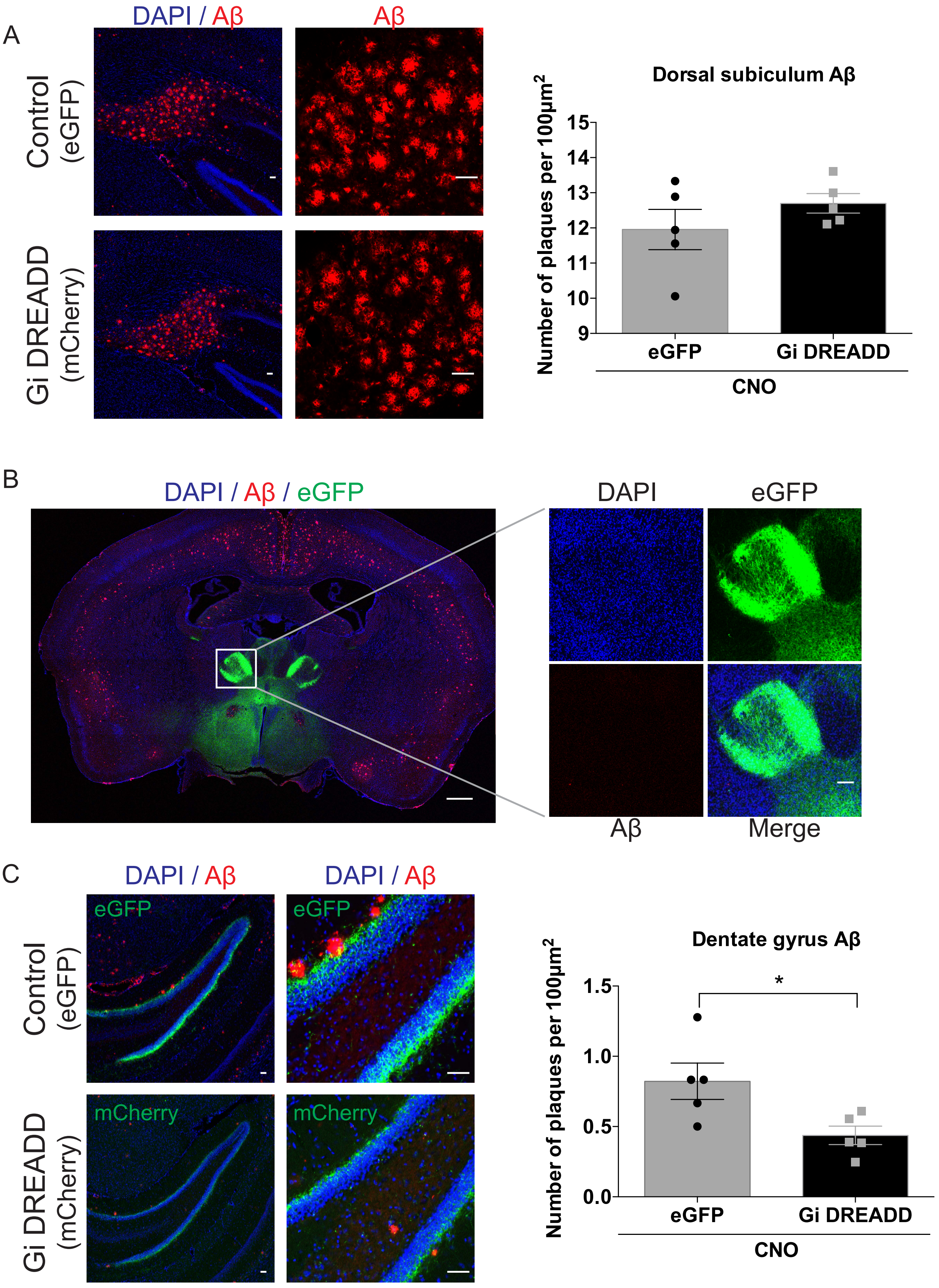

Supplementary Figure 3: Chronically inhibiting activity leads to a reduction of amyloid plaque in the downstream brain region

A) Images (left) and quantification (right) of Aβ plaque (red) in the dorsal subiculum of control and Gi DREADD mice. DAPI (blue). Scale bar 50μm. Unpaired Student's t-test; $t_{(8)}$ = 1.173, p = 0.2745. N= 5 mice per group. Graph reports mean ± standard error.
B) A coronal section image (left) showing DAPI (blue), Aβ plaque (red), and eGFP-labelled axons (green) in the anterior thalamus of 5XFAD mice. Scale bar 500μm. Enlarged images (right) showing that Aβ plaque (red) is non-observable in the anterior thalamic nuclei (green). DAPI (blue). Scale bar 100μm.
C) Images (left) and quantification (right) of Aβ plaque (red) in the dentate gyrus of control and Gi DREADD mice. DAPI (blue), eGFP (green in control group), and mCherry (green in mCherry group). Scale bar 50μm. Unpaired Student's t-test; $t_{(8)}$ = 2.653, p = 0.0291. *p<0.05. N= 5 mice per group. Graph reports mean ± standard error.

Supplementary Table 1

Human Specimen Donor Table - Netherlands Brain Bank						
Patient	Sex	Age	Clinical Information		Mammillary Body Amyloid Summary	
			CDR	Braak	Histology Count	SWITCH Count
All individuals						
1	male	70	0	0	0	0
2	male	80	0	2	7	21293
3	female	91	0	3	6	2050
4	male	74	AD	5	6	8443

Supplementary Table 1
Netherlands Brain Bank patient data summary table. Table includes sex and age, as well as clinical information provided the bank (Clinical dementia rating (CDR) and Braak and Braak Stage (Braak)), as well as summary data from the SWITCH and histology processing and whether grossly identified white matter tracts appeared to have amyloid deposits.

Supplementary Table 2

Human Specimen Donor Table - Religious Orders Study									
Patient	Sex	Cognitive Diagnosis	Pathology Scores			Tau Pathology Summary		Mammillary Body Amyloid Summary	
			CERAD	NIA-Reagan	Braak	NFT (silver stain)	Tangles (AT8)	Count	% Area
Cognitively healthy individuals									
1	male	None	2	2	4	0.65	1.31	69	0.021
2	male	None	3	3	3	0.26	3.98	355	0.396
3	male	None	1	2	4	0.56	5.80	304	0.967
4	male	None	2	2	3	0.16	1.19	48	0.029
5	male	None	4	4	0	0.00	0.03	259	0.101
6	female	None	4	3	3	0.35	2.93	426	0.174
7	female	None	3	3	1	0.03	0.83	10	0.048
8	female	None	2	3	0	0.00	0.00	31	0.01
9	female	None	1	2	4	0.25	3.16	63	0.129
10	female	None	1	2	4	0.62	7.83	1058	0.636
Cognitively diagnosed AD Patients									
11	male	AD	1	1	6	3.27	22.54	2154	3.096
12	female	AD	2	2	3	0.12	4.65	4109	2.749
13	female	AD	2	3	1	0.02	0.29	78	0.057
14	female	AD	1	1	5	0.83	27.60	2531	4.152
15	female	AD	2	2	5	2.58	10.99	1261	1.332
16	female	AD	2	2	4	0.26	3.94	101	0.017

Supplementary Table 2

Religious Orders Study patient data summary table. Table includes sex and clinical diagnosis, as well as pathology summary scores (CERAD, NIA-Reagan, Braak and Braak) and tau quantification summary data (silver stain identified NFT, AT8+ tangles) provided by ROS investigators. The table also includes well as summary data on the mammillary body amyloid load for each individual (Count, Percent Area, White Matter: N = no, Y = yes.

<u>Supplementary Methods</u>

SWITCH protocol for labeling intact mouse brain

Solutions:

SWITCH Fixative	1% glutaraldehyde<br>4% paraformaldehyde<br>in 1X PBS
Inactivation Solution	1% acrylamide<br>1M glycine<br>in 1X PBS
PBST	0.1% triton- X100<br>in 1X PBS
Clearing Solution (pH 8.5 - 9.0)	200mM sodium dodecylsulfate<br>20mM lithium hydroxide monohydrate<br>4mM boric acid<br>in ddH_2O
Blocking Buffer	2% bovine serum albumin<br>in PBST
Weak Binding Solution (pH 10.5)	275mM NaCl<br>5mM KCl<br>10mM Na_2HPO_4<br>0.02% sodium azide<br>in PBST
Reversal Buffer (pH 7.4)	37.75mM Na_2HPO_4<br>3.53mM KH_2PO_4<br>0.02% sodium azide<br>in PBST
Hoechst solution	1mg/ml Hoechst 33258<br>in ddH_2O
Refractive index matching solution (RIMS)	75g Histodenz<br>20mL 0.1M phosphate buffer<br>60mL ddH_2O

Abbreviations:

m, h, d	minutes, hours, days
C	degrees Celcius

SWITCH protocol for labeling intact mouse brain

Pre-steps if clearing:

I. Perfuse with ice-cold PBS immeidately followed by SWITCH fixative.
II. Post-fix brains in SWITCH fixative for 72h at 4C on rocker.
III. Inactivate brains by incubating in inactivation solution for 48h at room temperature.
IV. Wash brains 12h in PBST, shaking at room temperature.
V. (Cut whole-brains into hemispheres)[1]
VI. Clear brains by incubating in clearing solution at 37-55C until white matter tracts are translucent.
VII. Wash for 48h at room temperature in PBST.

SWITCH labeling (for hemispheres[2]):

I. Incubate sections in weak binding solution for 24h.
II. Put directly into 1mL[3] of weak binding solution and add concentrated primary.[4]
III. Leave shaking for at least 72 hours[5] at 37C.
IV. Calculate the volume of primary incubation solution. Add extra to account for the volume of solution in the tissue.[6]
V. Add ⅛ of the volume calculated in step IV of reversal buffer[7] each hour for 8 hours.
VI. After final addition, let samples incubate in primary for at least 12h at 37C.[8]
VII. Wash 24h in PBST, shaking at 37C.
VIII. Use a secondary antibody[9] at the same dilution as the primary antibody. Dilute in PBST, shaking at 37C for the same duration as step III.
IX. Wash 24h in 1XPBS, shaking at room temperature.
X. Incubate in Hoechst diluted 1:100 in 1XPBS to label nuclei for 12h at room temperature.
XI. Wash 48h in 1XPBS, shaking at room temperature.
XII. Incubate in RIMS for 24h at room temperature.
XIII. Mount samples for imaging.

---

[1] Hemispheres will clear more quickly, so if you plan to cut them, doing it in advance is advantageous. If not, brains can be cut into hemispheres or other large (imprecise) chunks after clearing.
[2] For whole brain, multiply weak binding incubation and secondary incubation by 2. Washes and other incubation times remain the same or should be empirically tested.
[3] Solution should just cover the tissue. Volume may be different for whole brain or depending on the tube.
[4] Primary should be concentrated into weak binding solution using **insert filter name.** NB: Not concentrating antibody will change the composition of the weak binding solution and it may not work as well.
[5] 72h is the absolute minimum for a hemisphere and is likely not enough time for most experiments. 5d is usually very good for a hemisphere. For whole brain, scale accordingly.
[6] For example, if you have 1mL weak binding + 25uL concentrated primary antibody + 250uL volume in sample = 1275uL
[7] For the hemisphere with 1275 total, you add 160ul every hour for 8 hours. The volume will scale with the size of the tissue.
[8] This is the only step where longer *isn't* always better. You want enough time for the ionic concentration to fully equalize and allow antibody binding, but you don't want to leave it too long or you will get strong signal on the surface. If you don't leave it long enough, you might get weak signal, especially if there wasn't much labeling during the weak binding incubation. This can also be done at 37C. The specifics for this step likely need to be empirically tested. It can be tested on smaller chunks and scaled.
[9] Use fragment AlexaFluor antibodies (Cell Signaling Technologies or AbCam).

SWITCH protocol for labeling mouse brain sections

Solutions:

SWITCH Fixative	1% glutaraldehyde<br>4% paraformaldehyde<br>in 1X PBS
Inactivation Solution	1% acrylamide<br>1M glycine<br>in 1X PBS
PBST	0.1% triton- X100<br>in 1X PBS
Clearing Solution (pH 8.5 - 9.0)	200mM sodium dodecylsulfate<br>20mM lithium hydroxide monohydrate<br>4mM boric acid<br>in ddH_2O
Blocking Buffer	2% bovine serum albumin<br>in PBST
Weak Binding Solution (pH 10.5)	275mM NaCl<br>5mM KCl<br>10mM Na_2HPO_4<br>0.02% sodium azide<br>in PBST
Reversal Buffer (pH 7.4)	37.75mM Na_2HPO_4<br>3.53mM KH_2PO_4<br>0.02% sodium azide<br>in PBST
Hoechst solution	1mg/ml Hoechst 33258<br>in ddH_2O
Refractive index matching solution (RIMS)	75g Histodenz<br>20mL 0.1M phosphate buffer<br>60mL ddH_2O

Abbreviations:

m, h, d	minutes, hours, days
C	degrees Celcius

SWITCH protocol for labeling mouse brain sections

Pre-steps if clearing:

I. Perfusion with 1% glutaraldehyde, 4% paraformaldehyde
II. Inactivation in 1% acrylamide, 1M glycine in 1XPBS + 0.1% triton- X100
III. Wash 3 x 15m in 1X PBST, shaking at room temperature
IV. Clear at 37-55C until white matter tracts are translucent.
V. Wash for 1-12h at room temperature in 1XPBST.

SWITCH labeling (for 40um sections[1]):

I. Block in 2% BSA in 1XPBST for 2 hours at room temperature
II. Wash 3 x 15m in 1XPBST, shaking at room temperature
III. Incubate sections in weak binding buffer for 1 hour
IV. Put directly into 400uL[2] of primary diluted in weak binding buffer
V. Leave shaking at room temperature for at least 12 hours[3] at 37C[4]
VI. Calculate the volume of primary incubation solution plus a bit more for the volume of buffer that was in the tissue.
VII. Over 6 hours add □ of the volume calculated in step VI of reversal buffer[5]
VIII. After final addition, let samples incubate in primary for at least 1h at room temperature.[6]
IX. Wash 3 x 1h in 1XPBST, shaking at room temperature
X. Use a secondary antibody[7] at the same dilution as the primary antibody. Incubate in 1XPBST, shaking at room temperature for at least 4h.
XI. Wash 2 x 1h in 1XPBS, shaking at room temperature
XII. Incubate in 1:1000 in 1XPBS, 1mg/mL Hoechst to label nuclei for 1h at room temperature.
XIII. Wash 2 x 15m in 1XPBS, shaking at room temperature
XIV. Wash overnight in 1XPBS, shaking at room temperature
XV. (If clearing, incubate in RIMS for 2h at room temperature)
XVI. Mount samples for imaging

Comments

[1]for 100um sections multiply times by 4; for 250um sections multiply long incubations by 6 and shorter washes by 4
[2]for 100um 400ul + more concentrated antibody; for 250um sections likely 500uL - 750uL with concentrated antibody
[3]12 hours is the absolute minimum for 40um. for 100um, 24 hours is the minimum. for 250, 48 hours is the minimum. longer is always better with a sweet-spot between 48 and 72 hours
[4]if the antibody is very specific and/or lower affinity, then 38C will work very well. if the antibody is a bit dirty or very high affinity, you should leave it at room temperature or 4C
[5] for example, if you have 400uL, assume 425ul volume. add 71ul every hour for 6 hours. the volume and the time should be scaled for tissue thickness up to 8 hours for a whole brain.
[6] for 100um sections 2h minimum, for 250um 4h minimum. this is the only step where longer *isn't* always better. you want to get enough time for the ionic concentration to fully equalize and allow antibody binding, but you don't want to leave it too long or you will get strong signal on the surface. if you don't leave it long enough, you might get weak signal if there wasn't much labeling during the weak binding incubation. the timing on this step may need to be empirically tested.
[7] For 100um sections and larger, use fragment Alexafluor antibodies (I use from AbCam). For 40um sections, normal AlexaFluor antibodies from LifeTechnologies are best.

SWITCH protocol for labeling human autopsy specimens

Solutions:

SWITCH Fixative	1% glutaraldehyde<br>in 1X PBS
Inactivation Solution	1% acrylamide<br>1M glycine<br>in 1X PBS
PBST	0.1% triton- X100<br>in 1X PBS
Clearing Solution (pH 8.5 - 9.0)	200mM sodium dodecylsulfate<br>20mM lithium hydroxide monohydrate<br>4mM boric acid<br>in ddH_2O
Blocking Buffer	2% bovine serum albumin<br>in PBST
Weak Binding Solution (pH 10.5)	275mM NaCl<br>5mM KCl<br>10mM Na_2HPO_4<br>0.02% sodium azide<br>in PBST
Reversal Buffer (pH 7.4)	37.75mM Na_2HPO_4<br>3.53mM KH_2PO_4<br>0.02% sodium azide<br>in PBST
Hoechst solution	1mg/ml Hoechst 33258<br>in ddH_2O
Refractive index matching solution (RIMS)	75g Histodenz<br>20mL 0.1M phosphate buffer<br>60mL ddH_2O

Abbreviations:

m, h, d	minutes, hours, days
C	degrees Celcius

SWITCH protocol for labeling human autopsy specimens

Pre-steps if paraffin embedded:

I. Place blocks into Xylene for 8d. Switch Xylene solution twice per day.
II. Rehydrate blocks[1]:

Chemical	Timing
100% Ethanol	1 hour 20 minutes
100% Ethanol	1 hour 20 minutes
100% Ethanol	1 hour 20 minutes
95% Ethanol	1 hour
95% Ethanol	1 hour
Running dH_2O from tap	10 minutes
dH_2O	Storage

Pre-steps if clearing:

I. Post-fix tissue in SWITCH fixative for 10d at 4C on rocker.
II. Inactivate tissue by incubating in inactivation solution for 72h at room temperature.
III. Wash tissue 24h in PBST, shaking at room temperature.
IV. Clear brains by incubating in clearing solution at 37-55C until sample appears translucent.
V. Wash for 72h at room temperature in PBST.

SWITCH labeling:

I. Incubate tissue in weak binding solution for 24h.
II. Put directly into 5mL[2] of weak binding solution and add concentrated primary.[3]
III. Leave shaking for at least 5d[4] at 37C.
IV. Calculate the volume of primary incubation solution. Add extra for the volume in the tissue.[5]
V. Add ⅛ of the volume calculated in step IV of reversal buffer[6] each hour for 8 hours.
VI. After final addition, let samples incubate in primary for at least 12h at 37.[7]
VII. Wash 24h in PBST, shaking at 37C.

---

[1] This protocol works on paraffinized blocks that are 2-5mm thick. These steps should be tested depending on the brand of paraffin and the size of the blocks.

[2] Solution should just cover the tissue. Volume may be different depending on the tube or dish.

[3] Primary should be concentrated into weak binding solution using **insert filter name.** NB: Not concentrating antibody will change the composition of the weak binding solution and it may not work as well.

[4] 5d is the absolute minimum and is likely not enough time for most experiments. Test this timing empirically.

[5] For example, if you have 5mL weak binding + 25uL concentrated primary antibody + 500uL volume in sample = 5525uL

[6] For the hemisphere with 5525 total, you add 690uL every hour for 8 hours. The volume will scale with the size of the tissue.

[7] This is the only step where longer *isn't* always better. You want enough time for the ionic concentration to fully equalize and allow antibody binding, but you don't want to leave it too long or you will get strong signal on the surface. If you don't leave it long enough, you might get weak signal, especially if there wasn't much labeling during the weak binding incubation. This can also be done at 37C. The specifics for this step likely need to be empirically tested. It can be tested on smaller chunks and scaled.

SWITCH protocol for labeling human autopsy specimens

VIII. Use a secondary antibody[1] at the same dilution as the primary antibody. Dilute in PBST, shaking at 37C for the same duration as step III.
IX. Wash 24h in 1XPBS, shaking at room temperature.
X. Incubate in Hoechst diluted 1:100 in 1XPBS to label nuclei for 12h at room temperature.
XI. Wash 48h in 1XPBS, shaking at room temperature.
XII. Incubate in RIMS for 24h at room temperature.

[1] Use fragment AlexaFluor antibodies (Cell Signaling Technologies or AbCam).

Supplementary Citaitons

1. A. Bennett, D., A. Schneider, J., Arvanitakis, Z. & S. Wilson, R. Overview and Findings from the Religious Orders Study. *Curr. Alzheimer Res.* **9,** 628–645 (2012).
