# Supplementary material for: 3D mapping reveals network-specific amyloid progression and subcortical susceptibility in mice
Source: Commun Biol. 2019 Oct 4;2:360. doi: 10.1038/s42003-019-0599-8 (PMC6778135; doi:10.1038/s42003-019-0599-8)
Supplement: Supplementary file 2 — Description of Additional Supplementary Items [file 42003_2019_599_MOESM2_ESM.pdf]

Supplementary Movie Legends

Supplementary Movie 1: Whole brain amyloid labeling Representative movie of whole-brain amyloid labeling in a 12M aged 5XFAD mouse (female, dam). Labeling occurs homogenously throughout the brain including superficial areas like the cortex and deep regions within the thalamus. Brains were labeled using SWITCH protocols as described in the text with the D54D2 anti-amyloid beta antibody from Cell Signaling Technology, Inc.

Supplementary Movie 2: Amyloid progression with aging Representative movies of whole-hemisphere amyloid in A) 2 month, B) 4 month, C) 6 month, and D) 12 month aged 5XFAD mice (male). In the brains from the youngest individuals, amyloid aggregation occurs in highly specific regions. With age, the amyloid spreads to more areas and increases in density within the regions of initial deposition. Brains were labeled using SWITCH protocols as described in the text with the D54D2 anti-amyloid beta antibody from Cell Signaling Technology, Inc.

Supplementary Movie 3: Human mammillary body amyloid 3D rendering of the amyloid within mammillary bodies of individuals staged at A) Braak and Braak 0; B) Braak and Braak II, C) Braak and Braak III, and D) Braak and Braak V. In addition to changing amyloid density, a pattern of aggregation within the white matter, especially at stages II and III, emerges

Supplementary Movie 4: Hand annotation of regions according to white matter tracts Representative 3D rendering of the A) white matter tracts that can be used for whole-brain segmentation. B) Representative 3D view of the final annotation across the entire hemisphere. C) Final volume-rendered regions overlaid on the hemisphere for quantification.
